# Arteriosclerosis in the heart-brain axis and Alzheimer's disease plasma markers in the Rotterdam Study

**DOI:** 10.1177/13872877251372641

**Published:** 2025-09-03

**Authors:** Anna M Streiber, Julia Neitzel, Amber Yaqub, Meike W Vernooij, Daniel Bos

**Affiliations:** 1 Department of Radiology and Nuclear Medicine, 6993 Erasmus MC, Rotterdam, the Netherlands; 2 Department of Epidemiology, Erasmus MC, Rotterdam, the Netherlands; 3 Department of Epidemiology, 1857 Harvard T.H. Chan School of Public Health, Boston, MA, USA

**Keywords:** Alzheimer's disease, arteriosclerosis, biomarkers, cohort studies, neuroimaging

## Abstract

**Background:**

Arteriosclerosis in the heart-brain axis has emerged as an important area of study in Alzheimer's disease (AD) dementia research. While previous research primarily focused on structural brain changes, the relationship between arteriosclerosis and blood-based markers for AD dementia remains understudied.

**Objective:**

To comprehensively assess arteriosclerosis in the heart-brain axis and investigate its link to AD dementia plasma markers.

**Methods:**

Analyses are based on 2238 community-dwelling elderly from the population-based Rotterdam Study (52% female, mean age 69.5 ± 6.8 years) who underwent a non-contrast CT scan to quantify arteriosclerosis in the heart-brain axis. Coronary artery calcification, aortic arch calcification, extracranial carotid artery calcification, intracranial carotid artery calcification, and vertebrobasilar artery calcification were measured as proxies for arteriosclerosis. Participants had available total tau, neurofilament light chain (NfL), and amyloid-β (Aβ_40_, Aβ_42_, and Aβ_42/40_ ratio) plasma measures (median time difference between CT scan and plasma measurement 3.2 months). Using linear regression, we studied the relationship of calcification presence and burden in the abovementioned arteries with plasma markers.

**Results:**

A higher arteriosclerosis burden correlated with higher plasma marker concentrations, especially for NfL (e.g., β_intracranial carotid_: 0.15, [95% Confidence interval (CI): 0.04, 0.26]) and Aβ_40_ (e.g., β_intracranial carotid_: 0.18, [95% CI: 0.05, 0.30]). Additionally, vertebrobasilar artery calcification was most consistently associated with elevated levels of AD dementia plasma markers (e.g., total tau; β: 0.06. [95% CI: 0.01, 0.10]).

**Conclusions:**

Our findings emphasize the critical role of vascular health in neurodegenerative processes and underscore the importance of monitoring and managing arteriosclerosis.

## Introduction

Alzheimer's disease (AD) dementia exerts a significant burden on global health care systems, which is estimated to increase even further in the upcoming years.^1,2^ While the clinical symptoms of AD dementia are well documented, the underlying etiology requires further investigation.^3^ In this context, the heart-brain axis has emerged as a crucial area of study.^4^ Recent evidence suggests that arteriosclerosis within the heart-brain axis is associated with cognitive decline, smaller brain tissue volume, and cerebral small vessel disease.^5–8^ These pathological changes are hypothesized to be caused by impaired cerebral blood flow, inflammation, and endothelial dysfunction.

While much of the current research focuses on structural brain changes (e.g.,^7^), the relationship between arteriosclerosis and blood-based markers of AD dementia remains understudied. The development of blood-based biomarkers may eventually help to diagnose AD dementia in a non-invasive, cost-efficient manner.^9^ Beyond diagnosis, they also capture pathology earlier and more specifically than structural markers such as MRI, allowing researchers to explore the underlying etiology more comprehensively. Although some previous studies have investigated the link between subclinical atherosclerosis and selected plasma markers of neurodegeneration,^10,11^ no earlier study has comprehensively assessed arteriosclerosis throughout the heart-brain axis and, subsequently, linked it to multiple AD dementia plasma markers.

The current study aims to further elucidate the association between arteriosclerosis in the heart-brain axis and AD dementia plasma markers within the population-based Rotterdam Study. By investigating the relationship between vascular health and AD dementia biomarkers in a population-based setting, we hope to identify etiological factors underlying neurodegeneration that improve prevention of AD dementia. In this study, we will investigate the link between arteriosclerosis in five major arteries between the heart and the brain and plasma concentrations of total tau, neurofilament light chain (NfL), and amyloid-β peptides (Aβ_40_, Aβ_42_, and the Aβ_42/40_ ratio). All of these plasma markers have been previously linked to AD dementia and cognitive decline.^12,13^ While the Aβ_42/40_ ratio has been shown to predict AD dementia,^14^ its interpretation in an etiological context remains challenging.^15^ To provide a more nuanced overview, we also included Aβ_40_ and Aβ_42_ as separate measures.

## Methods

### Setting and study sample

**Figure 1.**
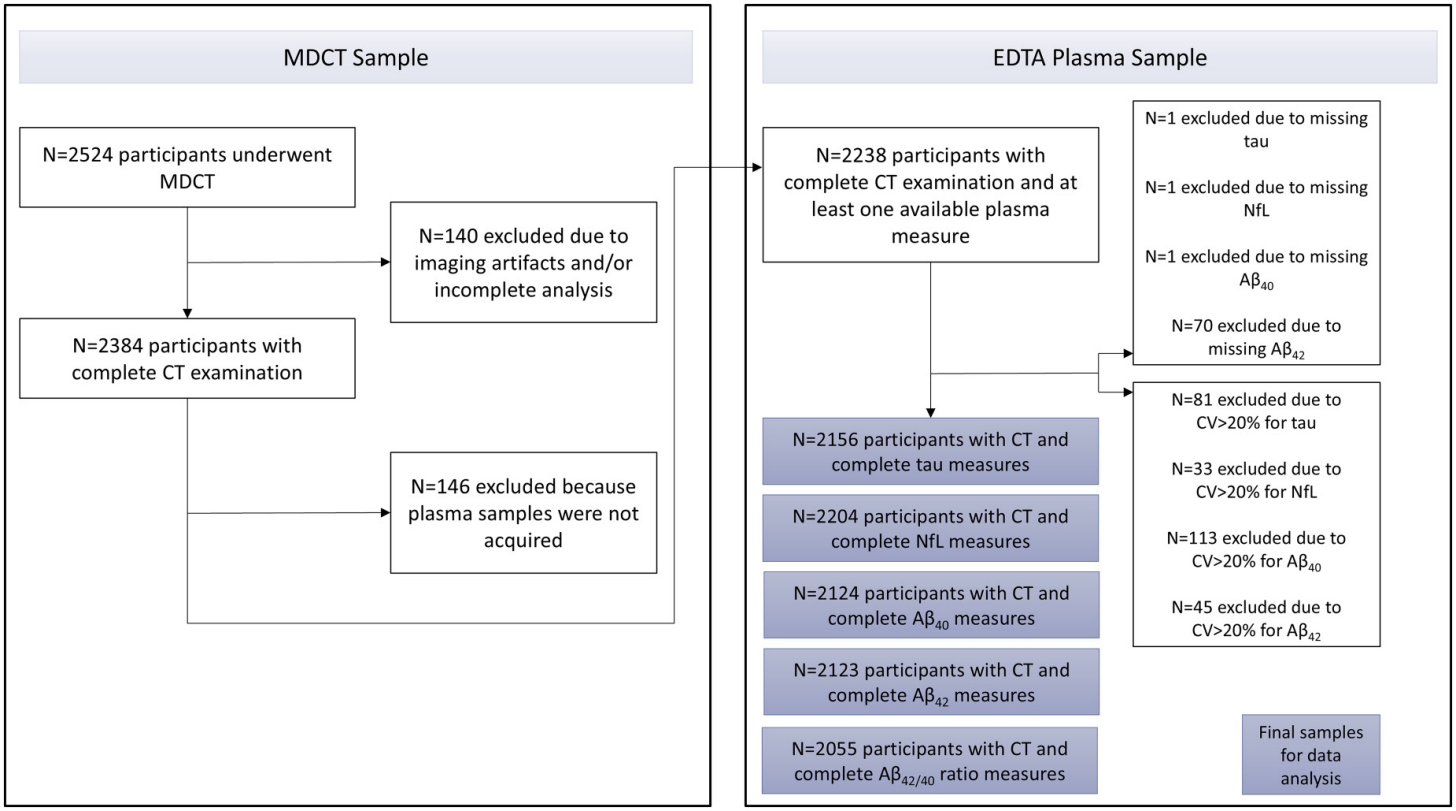
Flow chart showing the sample composition for this study. CV: coefficient variation.

The Rotterdam Study is a prospective cohort study that was implemented in 1990 to assess the prevalence and determinants of age-related diseases within the Ommoord district in the city of Rotterdam in The Netherlands.^16^ The Rotterdam Study is divided in four sub cohorts (RS-I, RS-II, RS-III, and RS-IV). At study entry, all participants receive a baseline measurement including a home interview followed by clinical examinations and plasma sampling at the research center. Every three to five years, participants are re-invited for a follow-up visit. Between 2003 and 2006, participants from the first and second study cohort were invited to undergo a non-contrast multidetector computed tomography (MDCT) scan to visualize extra- and intracranial arterial calcification. In total, 2524 participants (response rate 78%) were included of which 140 participants had to be excluded due to incomplete imaging data or imaging artifacts. Out of the participants who underwent the MDCT scan, 2156 had available tau measures, 2204 had complete NfL measures, 2124 had complete Aβ_40_ measures, 2123 had complete Aβ_42_ measures, and 2055 had complete Aβ_42/40_ ratio measures. A detailed overview of the sample selection is provided in Figure 1.

### Assessment of arterial calcification and plasma markers

*Calcification assessment.* As a hallmark of arteriosclerosis, we measured arterial calcification in five major arteries in the heart-brain axis, using non-contrast 16-slice or 64-slice MDCT scans (Somatom Sensation 16 or 64; Siemens, Forchheim, Germany). The field-of-view was optimized for visualization of blood vessels. The exact imaging parameters have been described elsewhere.^17^ We quantified coronary artery calcification, aortic arch calcification, and extracranial carotid artery calcification using commercially available software (Syngo CalciumScoring; Siemens). Intracranial carotid artery calcification and vertebrobasilar artery calcification were closely located to the scull base and, therefore, assessed employing a validated semi-automated scoring method.^18^ First, trained raters manually delineated arterial calcification from adjacent bone. Thereafter, calcification volume was calculated by multiplying the pixel number above the calcification threshold of 130 Hounsfield units with the pixel size and slice increment. The details of this scoring method have been extensively described previously.^19,20^

*Plasma marker assessment.* According to standard procedures, ethylenediamine tetra-acetic-treated plasma samples were collected between 2002 and 2005 and subsequently aliquoted and frozen at −80°C. Measurements (5094 samples) were obtained in two separate batches at Quanterix on a single molecule array (SiMoA^®^) HD-1 analyzer platform.^21^ The initial batch comprised 2000 samples, randomly chosen from 1000 participants of the fourth visit in RS-I and 1000 participants of the second visit in RS-II. The second batch consisted of 3094 samples, gathered from the remaining participants of these two study cohorts. NfL levels were quantified using the SiMoA NF-light^®^ advantage kit.^22,23^ To determine the concentrations of total tau, Aβ_40_, and Aβ_42_, the SiMoA Human Neurology 3-Plex A assay (N3PA) was used.^24^ Each sample was analyzed in duplicate and two quality control samples for each analyte were included on every plate. Plasma measures were excluded from the analyses if duplicates or individual measurements were missing, if the coefficient of variation for concentrations exceeded 20%, or if control samples fell outside the acceptable range.^12^

### Assessment of covariables

Covariables included age, sex, kidney function, *APOE* carriership (ε2, ε3, ε4), body mass index (BMI), hypertension, diabetes, dyslipidemia, smoking status (never, current, former), and alcohol intake (grams/day). Kidney function was assessed using estimated glomerular filtration rate (eGFR) measures, which are based on plasma creatinine.^25^ Kidney function was included as a covariate as it may affect the clearance of circulating proteins and the burden of arteriosclerosis.^26,27^ BMI was measured as weight (in kg)/height^2^ (in m). Hypertension was classified as present if one or more of the following characteristics were met: systolic blood pressure ≥ 140 mmHg, diastolic blood pressure ≥ 90 mmHg, use of antihypertensive medication. Participants were rated as diabetic if fasting serum glucose levels were ≥7 mmol/L and/or if they were using antidiabetic medication. Dyslipidemia was defined as using lipid-lowering medication and/or having a total cholesterol concentration of at least 6.2 mmol/L.

### Data analysis and statistics

All analyses were carried out using R version 4.2.2.^28^ Missing covariate data were imputed using multivariate imputation by chained equations^29^ with 30 imputations and 60 iterations. In the current RS round, eGFR was only assessed in a subset of participants (n = 965, 43.1%). Due to the large proportion of missing data, we used eGFR measures from the previous research visit as an auxiliary variable in the imputation model.

Determinants of plasma marker concentrations were calcification presence and calcification burden. Calcification data across all arteries were zero-inflated and, therefore, strongly skewed. To compute the calcification burden per individual, we temporarily excluded participants without calcification and assigned tertiles, representing low, medium, and high calcification, to the remaining participants. Subsequently, participants without calcification were added back to the dataset and received a score of zero. Importantly, these categories are data-driven and specific to the calcification distribution in our Rotterdam Study sample and should not be interpreted as clinical thresholds. Plasma data were log_2_-transformed to approximate a normal distribution and to reduce skewness. Subsequently, we standardized plasma data to improve comparability between the plasma markers, as they have inherently different ranges. Aβ_42/40_ ratio measures were only standardized and not log_2_-transformed, as the data followed a normal distribution.

In our main analyses, we first applied multiple linear regression to determine a relationship between calcification presence and standardized log_2_-transformed plasma biomarker concentrations. Subsequently, we studied the relationship between calcification burden (tertiles compared to no calcification) and plasma biomarker concentrations. Analyses were carried out for all possible combinations of arteries and plasma markers. All analyses were initially adjusted for age, sex, eGFR, and cohort (model 1). In a second step, we additionally adjusted for APOE genotype and cardiovascular risk factors (BMI, hypertension, diabetes, dyslipidemia, smoking status, alcohol intake; model 2). Regression estimates reflect the change in standardized plasma marker concentrations in log_2_ pg/mL based on calcification presence and burden compared to calcification absence. Due to the large number of missing eGFR data, we performed a sensitivity analysis excluding participants with incomplete eGFR assessments.

### Ethics statement

The Rotterdam Study has been approved by the Medical Ethics Committee of the Erasmus MC (registration number MEC 02.1015) and by the Dutch Ministry of Health, Welfare and Sport (Population Screening Act WBO, license number 1071272-159521-PG). The Rotterdam Study Personal Registration Data collection is filed with the Erasmus MC Data Protection Officer under registration number EMC1712001. The Rotterdam Study has been entered into the Netherlands National Trial Register (NTR; www.trialregister.nl) and into the WHO International Clinical Trials Registry Platform (ICTRP; https://apps.who.int/trialsearch/) under shared catalogue number NL6645 / NTR6831. All participants provided written informed consent to participate in the study and to have their information obtained from treating physicians.

## Results

### Descriptives and demographics

Approximately 52% of the participants were female and the mean age was 69.5 (*SD* *=* 6.8). The prevalence of coronary artery calcification, aortic arch calcification, as well as intra- and extracranial carotid artery calcification ranged primarily from 73.1% to 92.5%. The prevalence of vertebrobasilar calcification was notably lower, at 20.1%. A descriptive overview of sample characteristics is summarized in Table 1.

**Table 1.** Descriptive sample characteristics.

Variable	N = 2238
Cohort	
1	674 (30.1%)
2	1564 (69.9%)
Sex (female)	1168 (52.2%)
Age (y)	69.5(6.8)
Self-reported ethnic background	
North-West European	2015 (90.0%)
Other ethnic background	65 (2.9%)
Missing	158 (7.1%)
Time difference between CT scan and plasma measures (months)^a^	3.2 (0.1, 30.7)
BMI (kg/m²)	27.6 (4.0)
Missing	5 (0.2%)
Hypertension (yes)	1651 (73.8%)
Missing	2 (0.1%)
Dyslipidemia (yes)	648 (29.0%)
Missing	2 (0.1%)
Diabetes (yes)	202 (9.0%)
Missing	2 (0.1%)
Smoking	
Never	674 (30.1%)
Former	1222 (54.6%)
Current	284 (12.7%)
Missing	58 (2.6%)
Alcohol consumption (gr/day)	13.2 (15.2)
Missing	58 (2.6%)
eGFR	77.2 (14.4)
Missing	1273 (56.9%)
*APOE* Alleles	
*APOE* ε3	1255 (56.1%)
*APOE* ε2	272 (12.2%)
*APOE* ε4	585 (26.1%)
Missing	126 (5.6%)
Total tau^b^	2.4 (1.0)
NfL^b^	11.7 (6.6)
Aβ_40_^b^	249.5 (57.4)
Aβ_42_^b^	10.1 (2.9)
Aβ_42/40_	0.04 (0.01)
Coronary artery calcification prevalence	1832 (81.9%)
Coronary artery calcification volume^b^	49.5 (269.2)
Aortic arch calcification prevalence	2070 (92.5%)
Aortic arch calcification volume^b^	250 (783.5)
Extracranial carotid artery calcification prevalence	1635 (73.1%)
Extracranial carotid artery calcification volume^b^	22.1 (114.9)
Intracranial carotid artery calcification prevalence	1822 (81.4%)
Intracranial carotid artery calcification volume^b^	41.8 (129.7)
Vertebrobasilar artery calcification prevalence	450 (20.1%)
Vertebrobasilar artery calcification volume^c^	0, 598

Continuous values are reported as mean (SD). Categorical variables are reported as n (%).

^a^ Values are reported as median (minimum, maximum). There were 109 participants with a gap of more than 12 months between the CT and the plasma assessment.

^b^ Values are reported as median (IQR).

^c^ Values are reported as minimum, maximum.

### Relationship between calcification and plasma markers

**Figure 2.**
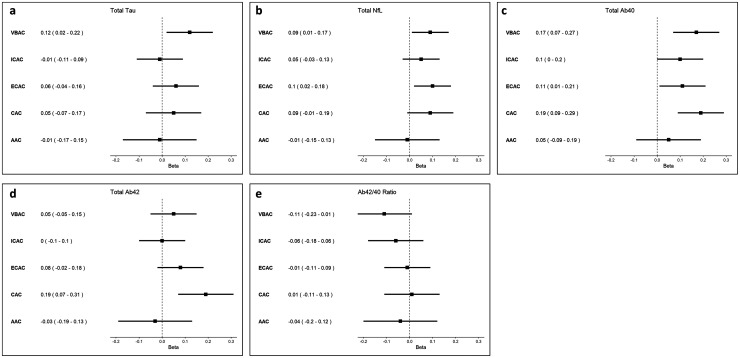
Forest plots showing linear regression estimates and associated 95% confidence intervals for the association between calcification presence and plasma marker concentrations. Beta reflects the change in standardized plasma marker concentrations (in log_2_ pg/mL) in participants with prevalent arterial calcification compared to participants without arterial calcification. VBAC: vertebrobasilar artery calcification; ICAC: intracranial carotid artery calcification; ECAC: extracranial carotid artery calcification; CAC: coronary artery calcification; AAC: aortic arch calcification.

**Figure 3.**
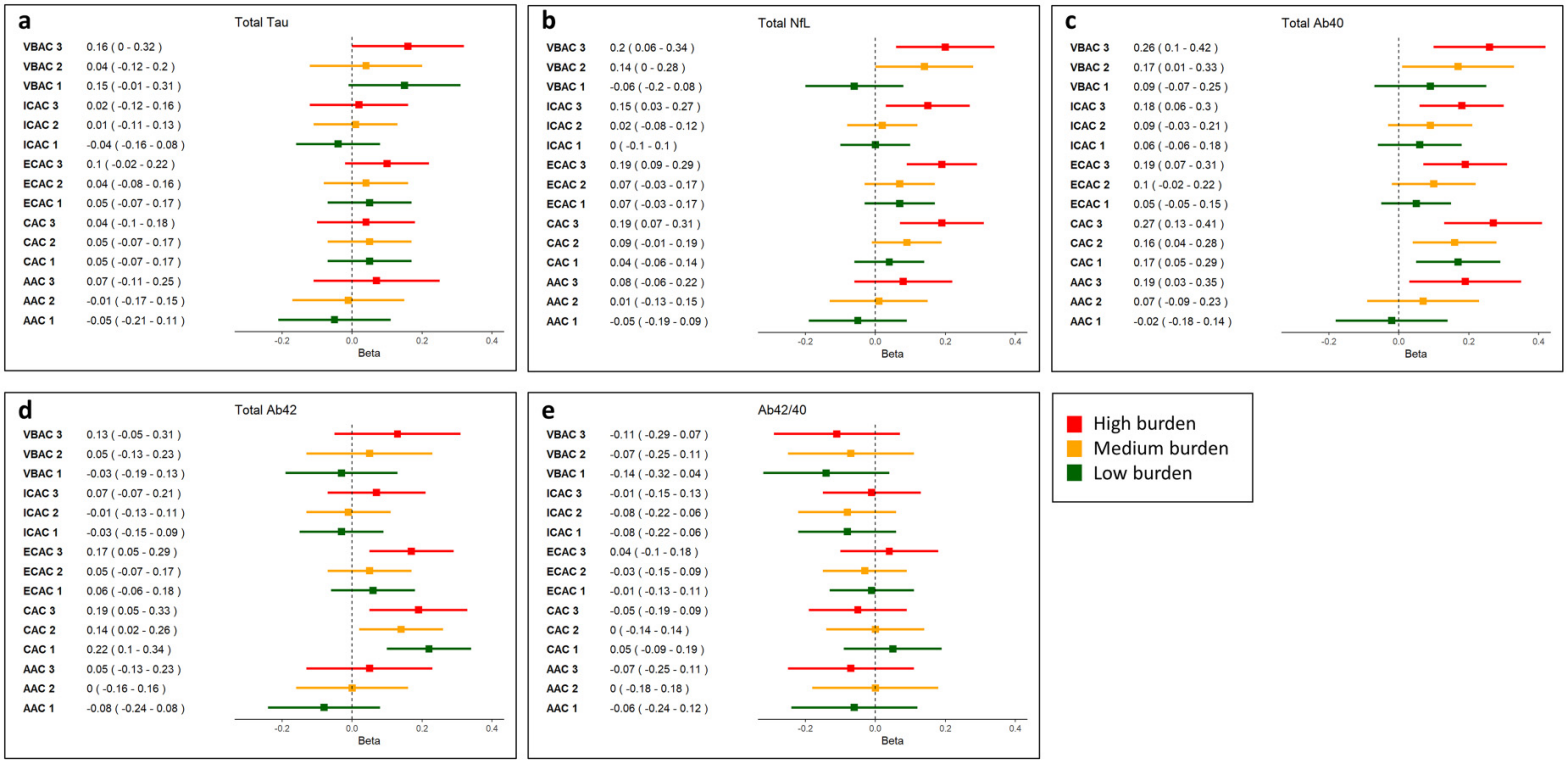
Forest plots showing linear regression estimates and associated 95% confidence intervals for the association between calcification burden (in tertiles) and plasma marker concentrations. Beta reflects the change in standardized plasma marker concentrations (in log_2_ pg/mL) in participants with a low (tertile 1), medium (tertile 2), and high (tertile 3) arterial calcification burden compared to participants without arterial calcification. VBAC: vertebrobasilar artery calcification; ICAC: intracranial carotid artery calcification; ECAC: extracranial carotid artery calcification; CAC: coronary artery calcification; AAC: aortic arch calcification.

The results of the fully adjusted regression analyses investigating the relationship of calcification presence and calcification burden with AD dementia plasma markers are presented in Figure 2 and Figure 3, respectively. The results of model 1 adjusted for age, sex, eGFR, and cohort are provided in Supplemental Table 1.

Vertebrobasilar artery calcification presence was most consistently associated with higher plasma concentrations of AD dementia markers. More specifically, it was associated with higher total tau (β: 0.06 [95% confidence interval (CI): 0.01–0.10]), higher NfL (β: 0.09 [95% CI: 0.00–0.17]), and higher Aβ_40_ concentrations (β: 0.17 [95% CI: 0.07–0.27]). Similarly, a higher vertebrobasilar artery calcification burden was linked to higher NFL (β_Tertile 2_: 0.14 [95% CI: 0.00–0.28]; β_Tertile 3_: 0.20 [95% CI: 0.06–0.34]), total tau (β_Tertile 3_: 0.08 [95% CI: 0.00–0.16]) and Aβ_40_ (β_Tertile 2_: 0.17 [95% CI: 0.02–0.33]; β_Tertile 3_: 0.26 [95% CI: 0.10–0.42]) concentrations.

While intracranial carotid artery calcification presence was solely associated with elevated Aβ_40_ concentrations (β: 0.10 [95% CI: 0.00–0.20]), a higher burden was associated with both higher Aβ_40_ (β_Tertile 3_: 0.18 [95% CI: 0.05–0.30]) and higher NfL concentrations (β_Tertile 3_: 0.15 [95% CI: 0.04–0.26]).

Presence of extracranial carotid artery calcification was associated with elevated NfL (β: 0.10 [95% CI: 0.03–0.18]) and Aβ_40_ (β: 0.11 [95% CI: 0.02–0.19]). The analyses focusing on extracranial carotid artery calcification burden indicated that a higher burden was associated with higher NfL (β_Tertile3_: 0.19 [95% CI: 0.09–0.30]), higher Aβ_40_ (β_Tertile 3_: 0.19 [95% CI: 0.08–0.31]), and higher Aβ_42_ concentrations (β_Tertile 3_: 0.17 [95% CI: 0.04–0.29]).

Presence of aortic arch calcification was not associated with higher biomarker concentrations across all plasma markers. However, a high aortic arch calcification burden was associated with higher Aβ_40_ concentrations (β_Tertile_ 3: 0.19 [95% CI: 0.03–0.35]).

Overall, coronary artery calcification presence was predominantly associated with elevated Aβ_40_ (β_presence_: 0.19 [95% CI: 0.09–0.29]) and Aβ_42_ concentrations (β_presence_: 0.19 [95% CI: 0.08–0.30]). This association remained for a low, medium, and high coronary artery calcification burden for both Aβ_42_ (β_Tertile1_: 0.22 [95% CI: 0.09–0.34]; β_Tertile2_: 0.14 [95% CI: 0.02–0.27]; β_Tertile3_: 0.19 [95% CI: 0.05–0.33]) and Aβ_40_ (β_Tertile1_: 0.18 [95% CI: 0.06–0.29]; β_Tertile2_: 0.19 [95% CI: 0.08–0.31]; β_Tertile3_: 0.31 [95% CI: 0.18–0.44]). Additionally, a high CAC burden was associated with higher NfL concentrations (β_Tertile 3_: 0.19 [95% CI: 0.07–0.30]).

To ensure that the imputation of the large number of missing eGFR values did not influence our results, we compared all clinical and demographic characteristics of participants with and without available eGFR measures. Subsequently, we repeated our regression analyses only in participants with complete eGFR data. The participant characteristics are presented in Supplemental Table 2, and the regression results are presented Supplemental Tables 3 and 4. Overall, the results were consistent between our study sample and the subgroup of participants with complete eGFR measures.

## Discussion

In this study among community-dwelling individuals, we assessed the relationship between arteriosclerosis in the heart-brain axis and AD dementia plasma markers. The prevalence of arteriosclerosis in this sample is comparable to earlier studies.^30–32^ Overall, a higher calcification burden along the heart-brain axis was associated with higher plasma marker concentrations, specifically NfL and Aβ_40_. Among the examined arteries, vertebrobasilar artery calcification presence and burden were specifically associated with elevated total tau, NfL, and Aβ_40_.

The association of vertebrobasilar artery calcification with various neurodegeneration markers could be partly explained by hemodynamic changes. Previous research indicates that intracranial arteriosclerosis is associated with changes in cerebral perfusion, including blood flow velocity and pulsatility.^33^ Furthermore, potential hypoperfusion injury resulting from these changes may promote the accumulation of misfolded proteins and axonal injury.^34,35^ It is hypothesized that vascular lesions, in combination with cerebral hemodynamic changes, may impair glucose metabolism and enhance β-secretase expression, consequently enhancing tau and Aβ pathology.^36^ Notably, the vertebrobasilar arteries supply critical deep brain structures, including the thalamus, which may upregulate NfL release in response to lower perfusion.^37^ This posterior circulation-dependent vulnerability might explain the specific link between vertebrobasilar artery calcification and both NfL and tau.

Beyond the vertebrobasilar system, a medium and high arteriosclerosis burden were associated with higher NfL concentrations in all arteries except the aortic arch. Earlier work from the Rotterdam study showed that a higher intracranial arteriosclerosis burden accelerates the development of small vessel disease,^7^ which is, in turn, associated with higher plasma NfL concentrations.^38^ Moreover, coronary artery calcification may trigger inflammatory cascades,^39^ possibly contributing to an upregulation of cytokines and further axonal injury.^40^ Taken together, these findings point to a broader vascular pathway linking systemic arteriosclerosis to neurodegeneration.

Besides its relationship to NfL, arteriosclerosis across the heart-brain axis was consistently associated with higher plasma Aβ_40_ but not Aβ_42_ concentrations. Both Aβ_40_ and Aβ_42_ are misfolded proteins that result from dysfunctional cleavage of the amyloid-β protein precursor.^41^ While Aβ_42_ tends to form extracellular plaques in the brain parenchyma, Aβ_40_ is more strongly associated with cerebrovascular pathology.^42^ Previous studies have demonstrated that Aβ_40_ is associated with vascular aging through its pro-inflammatory and pro-atherosclerotic properties,^43^ which are expressed in both the heart and the brain. Although Aβ_40_ is not a core pathological hallmark of AD dementia, previous studies (e.g.,^44^) propose that it may act as a risk-modifying factor, influencing AD dementia pathology indirectly rather than serving as a primary etiological factor. Thus, the observed association between arteriosclerosis and Aβ_40_ suggests an interplay between arteriosclerosis and Aβ_40_ that may influence AD dementia vulnerability.

In contrast to Aβ_40_ concentrations, Aβ_42_ plasma levels were almost exclusively associated with coronary artery calcification presence, irrespective of the arteriosclerosis burden. Cerebral Aβ_42_ has been previously linked to AD dementia pathogenesis, yet its link to cardiovascular disease remains unclear.^41^ Recent studies indicate no direct causal relationship between coronary heart disease and cerebral amyloid deposition,^45^ implying that other shared mechanisms, such as inflammation, oxidative stress, or lipid metabolism may explain this overlap.^4^ Thus, future studies are needed to understand these potential mediators and to provide insights into the complex interplay between cardiovascular health and AD dementia.

In contrast to individual Aβ_40_ and Aβ_42_ peptides, there was no association between arteriosclerosis in any of the arteries in the heart-brain axis and the Aβ_42/40_ ratio. The Aβ_42/40_ ratio is a recognized marker for cerebral amyloid accumulation.^46^ Our findings of associations with both Aβ_40_ and Aβ_42_ individually suggest that arteriosclerosis may influence the processing or clearance of these peptides. Yet, the absence of an association with the Aβ_42/40_ ratio points to a limited role in amyloidogenic processes specific to AD dementia. This is in line with prior findings from the Rotterdam Study and the Atherosclerosis Risk in Communities Study, both of which found no association between arteriosclerosis and cerebral amyloid accumulation.^47,48^ Taken together, these findings suggest that arteriosclerosis may not drive cerebral amyloid accumulation per se, but may contribute to AD dementia through alternative pathways, such as those involving Aβ_40_ and NfL.

Overall, the effects of arteriosclerosis on Aβ_40_ and NfL were most pronounced in individuals within the highest calcification tertiles, suggesting a dose–response relationship in which greater calcification volume is associated with stronger effects on AD dementia plasma markers. These findings imply that in individuals who already exhibit arteriosclerosis, further progression may still be clinically relevant and potentially modifiable. Intervening to slow or halt the progression of arteriosclerosis could, therefore, help mitigate possible downstream effects on neurodegenerative processes, especially if implemented before extensive calcification has occurred. While direct evidence linking vascular interventions to improvements in AD dementia plasma markers is limited, studies have demonstrated that addressing vascular risk factors can reduce cognitive decline and dementia risk.^49^ These findings support the notion that targeting arteriosclerosis may not only benefit cardiovascular health but could also delay or reduce neurodegenerative changes.

### Strengths and limitations

Strengths of our study include the comprehensive investigation of arteriosclerosis in the heart-brain axis and the population-based design, which allows for the identification of etiological mechanisms underlying AD dementia. However, potential limitations of our study need to be addressed. First, previous research indicates that, compared to total tau, phosphorylated tau has higher prognostic utility regarding clinical outcomes of AD dementia.^50,51^ Total tau is a rather broad, aspecific marker that is not exclusively associated with dementia.^52^ At the time of biomarker assessment, tau assessments were not yet widely implemented^53^ and only recently, the research focus shifted to more dementia-specific phosphorylated tau markers.^54^ Nonetheless, studies indicate that total tau can still provide added prognostic value, especially if combined with other biomarkers.^55^

Second, our study offers preliminary evidence on the relationship between arteriosclerosis and AD dementia plasma markers, yet it does not allow to establish a causal relationship. In order to establish causality in cross-sectional etiological studies, it is important to capture the time window in which relevant etiological changes occur.^56^ However, this time window may span decades and still remains incompletely understood,^57^ making it challenging to assess in our study. We explored the feasibility of applying causal inference methods, such as Mendelian randomization. However, this approach was not suitable due to sample overlap between our dataset and available genome-wide association studies,^58^ which could introduce bias. Moreover, many of our exposures and outcomes share common genetic loci, challenging the assumption of no horizontal pleiotropy. Given these limitations, we conclude that longitudinal studies are needed to unravel the temporal dynamics of these markers, facilitating causal research in the future.

Third, we cannot rule out residual confounding. For example, we were unable to adjust for physical activity, an established factor associated with both arteriosclerosis and neurodegeneration,^59^ due to data unavailability in the current sample. Nonetheless, we accounted for a broad range of relevant confounders, strengthening the internal validity of our findings. Future studies should consider incorporating measures of physical activity to further refine the robustness of the findings. Finally, dividing the calcification data into tertiles resulted in relatively small subgroups for our analyses, particularly for vertebrobasilar artery calcification, which had a low prevalence. This small sample size may have resulted in a lack of statistical power and, hence, an underestimation of the true effects associated with calcification tertiles.

### Conclusion

Our study indicates distinct pathways through which arteriosclerosis may contribute to the development of AD dementia. First, systemic arteriosclerosis (i.e., within the heart and brain) was associated with upregulated NfL, indicating that it may contribute to AD dementia through axonal damage. Second, intracranial arteriosclerosis in the vertebrobasilar arteries was additionally associated with higher total tau accumulation, suggesting a separate, posterior circulation-dependent mechanism. Third, we observed consistent associations between arteriosclerosis and plasma Aβ_40_ levels, indicating a vascular contribution to systemic amyloid dysregulation, which may serve as a risk factor for AD dementia. These findings highlight the importance of vascular health in multiple neurodegenerative processes relevant to AD dementia. Ultimately, targeted interventions on cardiovascular risk factors may offer a promising strategy to reduce the risk of AD dementia-related pathology.

## Supplemental Material

sj-docx-1-alz-10.1177_13872877251372641 - Supplemental material for Arteriosclerosis in the heart-brain axis and Alzheimer's disease plasma markers in the Rotterdam Study Supplemental material, sj-docx-1-alz-10.1177_13872877251372641 for Arteriosclerosis in the heart-brain axis and Alzheimer's disease plasma markers in the Rotterdam Study by Anna M Streiber, Julia Neitzel, Amber Yaqub, Meike W Vernooij and Daniel Bos in Journal of Alzheimer's Disease
