# Supplementary material for: Arteriosclerosis in the heart-brain axis and Alzheimer's disease plasma markers in the Rotterdam Study
Source: J Alzheimers Dis. 2025 Sep 3;107(4):1741–50. doi: 10.1177/13872877251372641 (PMC12495109; doi:10.1177/13872877251372641)
Supplement: sj-docx-1-alz-10.1177_13872877251372641 - Supplemental material for Arteriosclerosis in the heart-brain axis and Alzheimer's disease plasma markers in the Rotterdam Study [file sj-docx-1-alz-10.1177_13872877251372641.docx]

**Supplemental Material**

**Arteriosclerosis in the heart-brain axis and Alzheimer’s disease plasma markers in the Rotterdam Study**

**Supplemental Table 1.** Regression results of Model 1 adjusted for age, sex, cohort, and eGFR.

|  | Total tau | | NfL | | | Aβ_40_ | | | Aβ_42_ | | | Aβ_42/40_ | |
| --- | --- | --- | --- | --- | --- | --- | --- | --- | --- | --- | --- | --- | --- |
|  | Estimate | 95% CI | Estimate | 95% CI | Estimate | | 95% CI | Estimate | | 95% CI | Estimate | | 95% CI |
| CAC |  |  |  |  |  | |  |  | |  |  | |  |
| Presence | 0.07 | -0.03, 0.18 | 0.06 | -0.03, 0.16 | 0.21 | | 0.11, 0.31 | 0.18 | | 0.07, 0.29 | 0.00 | | -0.12, 0.12 |
| Low burden | 0.07 | -0.05, 0.20 | 0.01 | -0.10, 0.11 | 0.18 | | 0.06, 0.29 | 0.22 | | 0.10, 0.34 | 0.06 | | -0.07, 0.19 |
| Medium burden | 0.08 | -0.05, 0.21 | 0.06 | -0.04, 0.17 | 0.19 | | 0.07, 0.31 | 0.14 | | 0.01, 0.26 | -0.02 | | -0.16, 0.11 |
| High burden | 0.06 | -0.07, 0.20 | 0.16 | 0.04, 0.27 | 0.30 | | 0.17, 0.43 | 0.18 | | 0.04, 0.31 | -0.08 | | -0.22, 0.07 |
| AAC |  |  |  |  |  | |  |  | |  |  | |  |
| Presence | 0.00 | -0.15, 0.15 | -0.02 | -0.15, 0.12 | 0.07 | | -0.07, 0.22 | -0.03 | | -0.18, 0.12 | -0.06 | | -0.22, 0.10 |
| Low burden | -0.04 | -0.20, 0.12 | -0.06 | -0.20, 0.08 | -0.01 | | -0.16, 0.14 | -0.08 | | -0.24, 0.09 | -0.07 | | -0.24, 0.10 |
| Medium burden | 0.00 | -0.16, 0.16 | -0.01 | -0.15, 0.13 | 0.09 | | -0.07, 0.24 | -0.01 | | -0.18, 0.15 | -0.02 | | -0.20, 0.15 |
| High burden | 0.08 | -0.09, 0.25 | 0.06 | -0.08, 0.21 | 0.23 | | 0.07, 0.39 | 0.04 | | -0.14, 0.21 | -0.11 | | -0.29, 0.07 |
| ECAC |  |  |  |  |  | |  |  | |  |  | |  |
| Presence | 0.07 | -0.02, 0.17 | 0.10 | 0.02, 0.18 | 0.13 | | 0.04, 0.21 | 0.07 | | -0.03, 0.16 | -0.04 | | -0.14, 0.06 |
| Low burden | 0.06 | -0.05, 0.17 | 0.06 | -0.04, 0.15 | 0.06 | | -0.04, 0.17 | 0.04 | | -0.07, 0.15 | -0.04 | | -0.16, 0.08 |
| Medium burden | 0.05 | -0.06, 0.17 | 0.06 | -0.04, 0.16 | 0.12 | | 0.01, 0.23 | 0.03 | | -0.09, 0.14 | -0.07 | | -0.19, 0.06 |
| High burden | 0.11 | -0.01, 0.23 | 0.19 | 0.09, 0.29 | 0.23 | | 0.11, 0.34 | 0.14 | | 0.02, 0.27 | -0.01 | | -0.13, 0.12 |
| ICAC |  |  |  |  |  | |  |  | |  |  | |  |
| Presence | 0.00 | -0.11, 0.10 | 0.05 | -0.04, 0.14 | 0.11 | | 0.02, 0.21 | 0.00 | | -0.11, 0.10 | -0.08 | | -0.20, 0.03 |
| Low burden | -0.05 | -0.17, 0.08 | 0.01 | -0.09, 0.11 | 0.06 | | -0.05, 0.18 | -0.03 | | -0.15, 0.09 | -0.08 | | -0.21, 0.05 |
| Medium burden | 0.02 | -0.10, 0.14 | 0.01 | -0.10, 0.11 | 0.10 | | -0.01, 0.22 | -0.02 | | -0.14, 0.10 | -0.11 | | -0.24, 0.02 |
| High burden | 0.03 | -0.09, 0.16 | 0.16 | 0.05, 0.27 | 0.21 | | 0.09, 0.33 | 0.06 | | -0.07, 0.19 | -0.05 | | -0.19, 0.08 |
| VBAC |  |  |  |  |  | |  |  | |  |  | |  |
| Presence | 0.13 | 0.03, 0.23 | 0.09 | 0.00, 0.18 | 0.19 | | 0.09, 0.29 | 0.04 | | -0.07, 0.15 | -0.13 | | -0.24, -0.02 |
| Low burden | 0.17 | 0.01, 0.32 | -0.06 | -0.20, 0.08 | 0.10 | | -0.05, 0.25 | -0.04 | | -0.21, 0.12 | -0.17 | | -0.34, 0.00 |
| Medium burden | 0.04 | -0.12, 0.21 | 0.15 | 0.00, 0.29 | 0.19 | | 0.04, 0.35 | 0.05 | | -0.12, 0.22 | -0.09 | | -0.27, 0.09 |
| High burden | 0.18 | 0.02, 0.35 | 0.21 | 0.06, 0.35 | 0.28 | | 0.12, 0.43 | 0.12 | | -0.05, 0.29 | -0.13 | | -0.30, 0.05 |
| Estimate: change in standardized plasma marker concentrations in log2 pg/mL based on calcification presence and burden compared to no calcification. 95% CI: 95% confidence interval. Low, medium, and high burden refer to the first, second, and third calcification tertile respectively. All estimates were compared to no calcification. CAC: coronary artery calcification; AAC: aortic arch calcification; ECAC: extracranial carotid artery calcification; ICAC: Intracranial carotid artery calcification; VBAC: vertebrobasilar artery calcification | | | | | | | | | | | | | |

**Supplemental Table 2.** Demographic and clinical characteristics of participants with and without eGFR measures

|  | Missing eGFR data | Available eGFR data |  |
| --- | --- | --- | --- |
| Variable | N=1273 | N=965 |  |
| Cohort |  |  |  |
| 1 | 447 (35.1%) | 227 (23.5%) |  |
| 2 | 826 (64.9%) | 738 (76.5%) |  |
| Sex (female) | 657 (51.6%) | 511 (53.0%) |  |
| Age (y) | 69.7 (6.69) | 69.3 (6.80) |  |
| BMI (kg/m²) | 27.5 (3.86) | 27.8 (4.04) |  |
| Missing | 4 (0.3%) | 1 (0.1%) |  |
| Hypertension (yes) | 947 (74.4%) | 704 (73.0%) |  |
| Missing | 2 (0.2%) | 0 (0%) |  |
| Dyslipidemia (yes) | 370 (29.1%) | 278 (28.8%) |  |
| Missing | 2 (0.2%) | 0 (0%) |  |
| Diabetes (yes) | 117 (9.2%) | 85 (8.8%) |  |
| Missing | 2 (0.2%) | 0 (0%) |  |
| Smoking |  |  |  |
| Never | 386 (30.3%) | 288 (29.8%) |  |
| Former | 678 (53.3%) | 544 (56.4%) |  |
| Current | 167 (13.1%) | 117 (12.1%) |  |
| Missing | 42 (3.3%) | 16 (1.7%) |  |
| Alcohol consumption (gr/day) | 13.2 (15.1) | 13.3 (15.2) |  |
| Missing | 42 (3.3%) | 16 (1.7%) |  |
| *APOE* Alleles |  |  |  |
| *APOE* ε3 | 703 (55.2%) | 552 (57.2%) |  |
| *APOE* ε2 | 157 (12.3%) | 115 (11.9%) |  |
| *APOE* ε4 | 342 (26.9%) | 243 (25.2%) |  |
| Missing | 71 (5.6%) | 55 (5.7%) |  |
| Total tau^a^ | 2.59 (1.09) | 2.15 (0.873) |  |
| NfL^a^ | 11.4 (6.29) | 12.2 (6.95) |  |
| Aβ_40_^a^ | 248 (57.7) | 250 (56.6) |  |
| Aβ_42_^a^ | 9.31 (2.44) | 11.3 (2.93) |  |
| Aβ_42/40_ | 0.04 (0.01) | 0.05 (0.01) |  |
| Coronary artery calcification prevalence | 1044 (82.0%) | 788 (81.7%) |  |
| Coronary artery calcification volume^a^ | 56.1 (264) | 41.9 (271) |  |
| Aortic arch calcification prevalence | 1185 (93.1%) | 885 (91.7%) |  |
| Aortic arch calcification volume^a^ | 260 (826) | 228 (749) |  |
| Extracranial carotid artery calcification prevalence | 941 (73.9%) | 694 (71.9%) |  |
| Extracranial carotid artery calcification volume^a^ | 24.3 (114) | 19.2 (117) |  |
| Intracranial carotid artery calcification prevalence | 1034 (81.2%) | 788 (81.7%) |  |
| Intracranial carotid artery calcification volume^a^ | 44.2 (134) | 39.9 (121) |  |
| Vertebrobasilar artery calcification prevalence | 275 (21.6%) | 175 (18.1%) |  |
| Vertebrobasilar artery calcification volume^b^ | 0, 598 | 0, 505 |  |
| Continuous values are reported as mean (SD). Categorical variables are reported as n (%).  ^a^ Values are reported as median (IQR)  ^b^ Values are reported as minimum, maximum | | | |

**Supplemental Table 3.** Regression results including only participants with available eGFR measures (model 1 adjusted for age, sex, cohort, and eGFR).

|  | Total tau | | | NfL | | | Aβ_40_ | | | Aβ_42_ | | | Aβ_42/40_ | |  |
| --- | --- | --- | --- | --- | --- | --- | --- | --- | --- | --- | --- | --- | --- | --- | --- |
|  | Estimate | 95% CI | Estimate | | 95% CI | Estimate | | 95% CI | Estimate | | 95% CI | Estimate | | 95% CI |  |
| CAC |  |  |  | |  |  | |  |  | |  |  | |  |  |
| Presence | 0.04 | -0.12, 0.19 | -0.02 | | -0.16, 0.12 | 0.25 | | 0.11, 0.40 | 0.21 | | 0.05, 0.36 | 0.00 | | -0.17, 0.17 |  |
| Low burden | 0.06 | -0.12, 0.23 | -0.02 | | -0.17, 0.14 | 0.22 | | 0.06, 0.38 | 0.24 | | 0.07, 0.41 | 0.06 | | -0.13, 0.25 |  |
| Medium burden | 0.01 | -0.17, 0.19 | -0.05 | | -0.21, 0.11 | 0.24 | | 0.07, 0.40 | 0.19 | | 0.02, 0.37 | 0.02 | | -0.18, 0.22 |  |
| High burden | 0.03 | -0.17, 0.22 | 0.01 | | -0.16, 0.18 | 0.34 | | 0.16, 0.52 | 0.15 | | -0.05, 0.34 | -0.16 | | -0.38, 0.05 |  |
| AAC |  |  |  | |  |  | |  |  | |  |  | |  |  |
| Presence | 0.09 | -0.12, 0.30 | 0.01 | | -0.18, 0.21 | 0.12 | | -0.08, 0.32 | 0.03 | | -0.17, 0.24 | -0.06 | | -0.28, 0.17 |  |
| Low burden | 0.04 | -0.19, 0.26 | -0.03 | | -0.23, 0.17 | 0.01 | | -0.19, 0.22 | -0.04 | | -0.26, 0.17 | -0.07 | | -0.31, 0.17 |  |
| Medium burden | 0.11 | -0.12, 0.35 | 0.03 | | -0.17, 0.24 | 0.17 | | -0.05, 0.38 | 0.09 | | -0.13, 0.32 | -0.02 | | -0.27, 0.23 |  |
| High burden | 0.17 | -0.07, 0.41 | 0.09 | | -0.13, 0.30 | 0.29 | | 0.06, 0.51 | 0.12 | | -0.11, 0.35 | -0.08 | | -0.34, 0.18 |  |
| ECAC |  |  |  | |  |  | |  |  | |  |  | |  |  |
| Presence | 0.12 | -0.01, 0.25 | 0.12 | | 0.00, 0.24 | 0.12 | | 0.00, 0.25 | 0.11 | | -0.02, 0.24 | 0.04 | | -0.11, 0.18 |  |
| Low burden | 0.14 | -0.02, 0.30 | 0.09 | | -0.06, 0.23 | 0.08 | | -0.07, 0.23 | 0.07 | | -0.09, 0.22 | 0.01 | | -0.16, 0.18 |  |
| Medium burden | 0.04 | -0.13, 0.21 | 0.09 | | -0.06, 0.24 | 0.11 | | -0.05, 0.27 | 0.17 | | 0.00, 0.33 | 0.14 | | -0.05, 0.32 |  |
| High burden | 0.18 | 0.01, 0.35 | 0.21 | | 0.06, 0.36 | 0.20 | | 0.04, 0.35 | 0.12 | | -0.05, 0.29 | -0.03 | | -0.22, 0.15 |  |
| ICAC |  |  |  | |  |  | |  |  | |  |  | |  |  |
| Presence | 0.01 | -0.14, 0.17 | -0.06 | | -0.20, 0.08 | 0.12 | | -0.02, 0.27 | 0.07 | | -0.07, 0.22 | -0.02 | | -0.19, 0.14 |  |
| Low burden | -0.04 | -0.21, 0.14 | -0.11 | | -0.27, 0.04 | 0.10 | | -0.06, 0.26 | 0.09 | | -0.08, 0.26 | -0.01 | | -0.20, 0.18 |  |
| Medium burden | 0.08 | -0.10, 0.25 | -0.10 | | -0.26, 0.05 | 0.09 | | -0.07, 0.25 | 0.03 | | -0.14, 0.20 | -0.06 | | -0.25, 0.13 |  |
| High burden | 0.01 | -0.18, 0.20 | 0.09 | | -0.08, 0.26 | 0.21 | | 0.04, 0.39 | 0.12 | | -0.07, 0.30 | 0.00 | | -0.21, 0.20 |  |
| VBAC |  |  |  | |  |  | |  |  | |  |  | |  |  |
| Presence | 0.19 | 0.04, 0.35 | 0.10 | | -0.04, 0.24 | 0.15 | | 0.01, 0.30 | 0.07 | | -0.09, 0.23 | -0.08 | | -0.25, 0.09 |  |
| Low burden | 0.31 | 0.07, 0.56 | -0.14 | | -0.36, 0.08 | 0.00 | | -0.23, 0.22 | -0.05 | | -0.31, 0.20 | -0.13 | | -0.41, 0.15 |  |
| Medium burden | 0.14 | -0.10, 0.37 | 0.22 | | 0.02, 0.43 | 0.18 | | -0.03, 0.40 | 0.01 | | -0.22, 0.25 | -0.08 | | -0.34, 0.18 |  |
| High burden | 0.13 | -0.13, 0.39 | 0.22 | | -0.01, 0.45 | 0.29 | | 0.05, 0.53 | 0.28 | | 0.02, 0.55 | -0.03 | | -0.32, 0.27 |  |
| Estimate = change in standardized plasma marker concentrations in log2 pg/mL based on calcification presence and burden compared to no calcification. 95% CI: 95% confidence interval. Low, medium, and high burden refer to the first, second, and third calcification tertile respectively. All estimates were compared to no calcification. CAC: coronary artery calcification; AAC: aortic arch calcification; ECAC: extracranial carotid artery calcification; ICAC: intracranial carotid artery calcification; VBAC: vertebrobasilar artery calcification | | | | | | | | | | | | | | | |

**Supplemental Table 4.** Regression results including only participants with available eGFR measures (model 2 adjusted for age, sex, cohort, eGFR, *APOE* genotypes, and cardiovascular risk factors).

|  | Total tau | | | NfL | | | Aβ_40_ | | | Aβ_42_ | | | Aβ_42/40_ | |  |
| --- | --- | --- | --- | --- | --- | --- | --- | --- | --- | --- | --- | --- | --- | --- | --- |
|  | Estimate | 95% CI | Estimate | | 95% CI | Estimate | | 95% CI | Estimate | | 95% CI | Estimate | | 95% CI |  |
| CAC |  |  |  | |  |  | |  |  | |  |  | |  |  |
| Presence | 0.01 | -0.14, 0.17 | -0.01 | | -0.14, 0.13 | 0.24 | | 0.09, 0.39 | 0.21 | | 0.06, 0.37 | 0.01 | | -0.16, 0.18 |  |
| Low burden | 0.03 | -0.14, 0.20 | 0.01 | | -0.14, 0.16 | 0.22 | | 0.06, 0.38 | 0.24 | | 0.07, 0.41 | 0.05 | | -0.13, 0.24 |  |
| Medium burden | -0.02 | -0.20, 0.17 | -0.04 | | -0.20, 0.11 | 0.22 | | 0.05, 0.39 | 0.20 | | 0.02, 0.38 | 0.03 | | -0.17, 0.23 |  |
| High burden | 0.01 | -0.18, 0.21 | 0.02 | | -0.15, 0.19 | 0.32 | | 0.14, 0.50 | 0.16 | | -0.04, 0.35 | -0.14 | | -0.36, 0.08 |  |
| AAC |  |  |  | |  |  | |  |  | |  |  | |  |  |
| Presence | 0.08 | -0.13, 0.29 | -0.02 | | -0.20, 0.17 | 0.10 | | -0.10, 0.30 | 0.06 | | -0.15, 0.26 | -0.01 | | -0.24, 0.22 |  |
| Low burden | 0.02 | -0.20, 0.24 | -0.05 | | -0.25, 0.14 | 0.01 | | -0.20, 0.22 | -0.17 | | -0.46, 0.11 | -0.03 | | -0.27, 0.21 |  |
| Medium burden | 0.12 | -0.11, 0.35 | -0.01 | | -0.21, 0.19 | 0.15 | | -0.07, 0.37 | -0.04 | | -0.32, 0.25 | 0.04 | | -0.21, 0.28 |  |
| High burden | 0.17 | -0.07, 0.41 | 0.06 | | -0.15, 0.27 | 0.25 | | 0.02, 0.48 | 0.01 | | -0.29, 0.30 | -0.02 | | -0.28, 0.24 |  |
| ECAC |  |  |  | |  |  | |  |  | |  |  | |  |  |
| Presence | 0.12 | -0.01, 0.26 | 0.11 | | -0.01, 0.23 | 0.10 | | -0.03, 0.22 | 0.14 | | 0.01, 0.28 | 0.10 | | -0.05, 0.24 |  |
| Low burden | 0.14 | -0.02, 0.30 | 0.09 | | -0.05, 0.23 | 0.06 | | -0.09, 0.21 | 0.10 | | -0.06, 0.25 | 0.06 | | -0.12, 0.23 |  |
| Medium burden | 0.05 | -0.12, 0.21 | 0.08 | | -0.07, 0.22 | 0.09 | | -0.07, 0.25 | 0.19 | | 0.02, 0.36 | 0.18 | | 0.00, 0.36 |  |
| High burden | 0.19 | 0.02, 0.37 | 0.20 | | 0.05, 0.35 | 0.15 | | -0.01, 0.31 | 0.16 | | -0.02, 0.33 | 0.06 | | -0.14, 0.25 |  |
| ICAC |  |  |  | |  |  | |  |  | |  |  | |  |  |
| Presence | 0.01 | -0.14, 0.16 | -0.07 | | -0.20, 0.07 | 0.10 | | -0.04, 0.25 | 0.09 | | -0.06, 0.24 | 0.00 | | -0.16, 0.17 |  |
| Low burden | -0.03 | -0.21, 0.14 | -0.11 | | -0.26, 0.04 | 0.09 | | -0.07, 0.25 | 0.09 | | -0.08, 0.26 | 0.01 | | -0.18, 0.19 |  |
| Medium burden | 0.06 | -0.11, 0.24 | -0.09 | | -0.25, 0.06 | 0.08 | | -0.09, 0.24 | 0.05 | | -0.12, 0.22 | -0.02 | | -0.21, 0.17 |  |
| High burden | 0.01 | -0.18, 0.20 | 0.08 | | -0.08, 0.25 | 0.17 | | -0.01, 0.35 | 0.14 | | -0.05, 0.33 | 0.04 | | -0.17, 0.25 |  |
| VBAC |  |  |  | |  |  | |  |  | |  |  | |  |  |
| Presence | 0.19 | 0.03, 0.34 | 0.09 | | -0.04, 0.23 | 0.14 | | 0.00, 0.28 | 0.08 | | -0.08, 0.24 | -0.05 | | -0.23, 0.12 |  |
| Low burden | 0.32 | 0.07, 0.56 | -0.16 | | -0.37, 0.05 | -0.01 | | -0.24, 0.22 | -0.05 | | -0.30, 0.20 | -0.13 | | -0.41, 0.15 |  |
| Medium burden | 0.13 | -0.10, 0.36 | 0.23 | | 0.03, 0.43 | 0.16 | | -0.06, 0.37 | 0.02 | | -0.22, 0.26 | -0.04 | | -0.30, 0.23 |  |
| High burden | 0.12 | -0.14, 0.38 | 0.20 | | -0.03, 0.43 | 0.29 | | 0.05, 0.53 | 0.31 | | 0.04, 0.58 | 0.01 | | -0.28, 0.31 |  |
| Estimate = change in standardized plasma marker concentrations in log2 pg/mL based on calcification presence and burden compared to no calcification. 95% CI: 95% confidence interval. Low, medium, and high burden refer to the first, second, and third calcification tertile respectively. All estimates were compared to no calcification. CAC: coronary artery calcification; AAC: aortic arch calcification; ECAC: extracranial carotid artery calcification; ICAC: intracranial carotid artery calcification; VBAC: vertebrobasilar artery calcification | | | | | | | | | | | | | | | |
